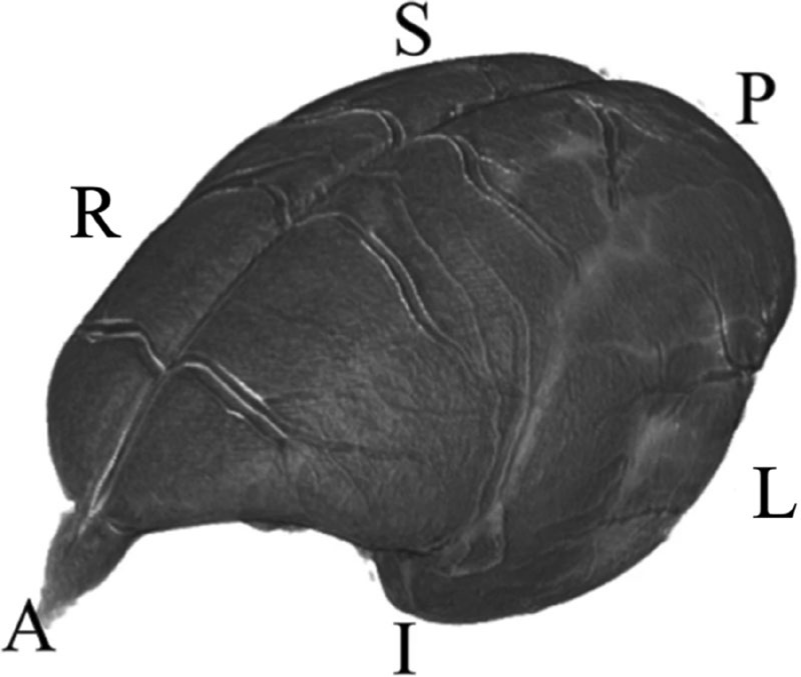# Author Correction: The Brain/MINDS 3D digital marmoset brain atlas

**DOI:** 10.1038/s41597-022-01247-z

**Published:** 2022-03-18

**Authors:** Alexander Woodward, Tsutomu Hashikawa, Masahide Maeda, Takaaki Kaneko, Keigo Hikishima, Atsushi Iriki, Hideyuki Okano, Yoko Yamaguchi

**Affiliations:** 1 Neuroinformatics Japan Center, RIKEN Brain Science Institute, Wako-shi, Saitama, 351-0198 Japan; 2 Laboratory for Symbolic Cognitive Development, RIKEN Brain Science Institute, Wako-shi, Saitama, 351-0198 Japan; 3 Laboratory for Marmoset Neural Architecture, RIKEN Brain Science Institute, Wako-shi, Saitama, 351-0198 Japan; 4 Animal Resources Section, Okinawa Institute of Science and Technology Graduate University, 1919-1 Tancha, Onna-son, Kunigami, Okinawa, 904-0495 Japan

Correction to: *Scientific Data* 10.1038/sdata.2018.9, published online 13 February 2018

In the original version of this Data Descriptor, the (R)ight and (L)eft labels in Figure 4 were incorrectly shown:
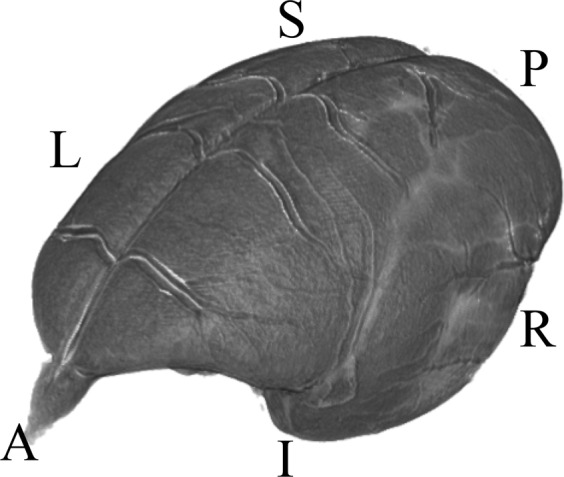


A corrected version has now been provided: